# New Approaches to Overcoming Antimicrobial Resistance in Endophthalmitis

**DOI:** 10.3390/ph17030321

**Published:** 2024-03-01

**Authors:** Noraliz Garcia O’Farrill, Mariana Abi Karam, Victor M. Villegas, Harry W. Flynn, Andrzej Grzybowski, Stephen G. Schwartz

**Affiliations:** 1 Department of Ophthalmology, University of Puerto Rico School of Medicine, San Juan, PR 00936, USA; noraliz.garcia@upr.edu (N.G.O.); victor.villegas@upr.edu (V.M.V.); 2 Department of Ophthalmology, MetroHealth, Cleveland, OH 44109, USA; abikarammariana@gmail.com; 3 Department of Ophthalmology and Visual Sciences, Case Western Reserve University School of Medicine, Cleveland, OH 44106, USA; 4 Department of Ophthalmology, Bascom Palmer Eye Institute, University of Miami Miller School of Medicine, Miami, FL 33136, USA; 5 Institute for Research in Ophthalmology, Foundation for Ophthalmology Development, 61-553 Poznan, Poland; ae.grzybowski@gmail.com

**Keywords:** endophthalmitis, cataract surgery prophylaxis, intracameral antibiotics, antimicrobial resistance, stewardship

## Abstract

Endophthalmitis is a rare but vision-threatening infection characterized by marked inflammation of intraocular fluids and tissues, uncommonly seen following surgery and intravitreal injection. Antimicrobials are used worldwide in the prophylaxis and treatment of bacterial and fungal infections of the eye and are standard treatment in the preoperative and postoperative care of surgical patients. However, antimicrobials are reported to be overprescribed in many parts of the world, which contributes to antimicrobial resistance (AMR). AMR complicates the prophylaxis and treatment of endophthalmitis. This article examines the prevalence and mechanisms of AMR in ocular microorganisms, emphasizing the importance of understanding AMR patterns for tailored treatments. It also explores prophylaxis and management strategies for endophthalmitis, with a discussion on the use of intracameral antibiotic administration. The use of prophylactic intracameral antibiotics during cataract surgery is common in many parts of the world but is still controversial in some locations, especially in the US. Finally, it highlights the role of stewardship in ophthalmology and its benefits in the treatment of endophthalmitis.

## 1. Introduction

Antimicrobials are used worldwide in the prophylaxis and treatment of bacterial and fungal infections of the eye and are standard treatment in the preoperative and postoperative care of surgical patients. However, antimicrobials are reported to be overprescribed in many parts of the world. For example, more than half of all antibiotic use in the United States (US) is described as “unnecessary”, per the US Centers for Disease Control and Prevention (CDC) [1].

The impact of antimicrobial resistance (AMR) in ophthalmology has been relatively underreported compared to other medical specialties. Within ophthalmology, perhaps the most severe infectious process is endophthalmitis. Endophthalmitis is a severe intraocular infection that can lead to severe visual impairment due to disruption of the blood–retinal barrier.

The pathophysiology of endophthalmitis involves microbial infection, either exogenous (following penetrating surgery, trauma, or corneal infection) or endogenous (caused by hematogenous spread from a distant source of infection). Infection within the eye generally results in severe intraocular inflammation. Disease severity is influenced by the infecting pathogen’s virulence (including bacterial load, replicative capacity, and toxin production) as well as the host’s immune response (involving inflammatory mediators and subsequent tissue necrosis) [2,3]. Angiogenesis also plays a role in the pathophysiology. There is a reported upregulation of vascular endothelial growth factor A (VEGF-A) in exogenous endophthalmitis, which promotes further blood–retinal barrier breakdown and exposes the tissues to injury by immune defense mechanisms [4]. 

Endophthalmitis is rare but remains a significant concern, especially in the context of very frequently performed procedures such as cataract surgery and intravitreal injections. Acute-onset postoperative endophthalmitis is typically defined as occurring within six weeks of intraocular surgery and is usually bacterial in origin. In addition, patients with endophthalmitis presenting more than 6 weeks following cataract surgery are categorized as chronic or delayed-onset postoperative endophthalmitis [5]. Broad-spectrum antimicrobials are essential tools used in ophthalmology and in the treatment of endophthalmitis, but emerging resistant organisms are an important threat to their continued efficacy [6]. Antimicrobial resistance may lead to poor clinical outcomes and increased costs, estimated to reach from USD 300 billion to over USD 1 trillion by 2050. In order to reduce the emergence of these resistant organisms, antibiotic stewardship programs seek to use antibiotics in a more targeted fashion [7].

The present manuscript reviews the features of postoperative endophthalmitis, including incidence rates, risk factors, prophylaxis, current treatment, and the challenges of AMR. Additionally, it highlights the role of antibiotic stewardship in ophthalmology, especially regarding the prophylaxis and treatment of endophthalmitis. 

## 2. Endophthalmitis: A Historical Perspective

The understanding of endophthalmitis and its management has evolved significantly. With the continued development of new antimicrobials and modes of medication delivery, along with the refinement of ocular surgeries, patient outcomes have greatly improved. Prior to the 1940s, before antibiotics, documented treatments encompassed a range of unconventional approaches which included the application of mercury oxycyanide, administration of anti-serum, application of localized heat, intravenous typhoid vaccine, and intramuscular injection of boiled milk. Outcomes improved with the introduction of systemic antimicrobial agents and improved further with the introduction of intravitreal antimicrobial therapy [8].

The Endophthalmitis Vitrectomy Study (EVS), published in 1995, was a randomized clinical trial (RCT) that recruited patients with acute-onset postoperative endophthalmitis following cataract surgery or secondary intraocular lens (IOL) implantation. Patients were randomized to receive vitreous tap and inject versus pars plana vitrectomy (PPV) as well as systemic antibiotic treatment (ceftazidime and amikacin) versus no systemic antibiotics. The investigators reported that, for patients with presenting visual acuity of hand motions or better, PPV and tap and inject were associated with similar outcomes. For patients with presenting visual acuity of light perception, PPV was associated with significantly more favorable outcomes. The use of systemic antibiotics was not associated with improved outcomes [9].

The EVS did not study patients with other categories of endophthalmitis, including chronic or delayed-onset postoperative endophthalmitis or endophthalmitis following intravitreal injections. Also, the EVS did not use more modern systemic antibiotics such as fluoroquinolones with greater ability to cross the blood–retinal barrier, resulting in increased vitreous concentration [10]. 

## 3. Acute-Onset Postoperative Endophthalmitis

The reported incidence of acute-onset postoperative endophthalmitis following cataract surgery is variable. A retrospective, cross-sectional analysis of more than 14 million surgeries reported an average rate of postoperative endophthalmitis within 90 days of cataract surgery of 1.30 per 1000 cataract surgeries (0.13%) for stand-alone cataract procedures among Medicare beneficiaries of 65 years and older between 2011 and 2019 [11]. Another similar study among Medicare beneficiaries that included various intraocular surgeries reported a 42-day postoperative endophthalmitis rate of 0.09%, and 0.08% for cataract surgeries alone [12]. Other series have reported rates between 0.063% and 0.20% [13,14,15,16].

Several risk factors have been identified for endophthalmitis. Demographic factors include older age (>75 years of age), ocular surface disease, or prior history of glaucoma or retinal surgery. Surgical risk factors include surgically complex cases (vitreous loss, posterior capsule rupture, need for anterior vitrectomy, prolonged surgical time) and cataract surgery combined with other procedures. Immediate sequential bilateral cataract surgery has not been identified as a separate risk factor [11,12,17,18,19,20].

## 4. Endophthalmitis after Intravitreal Injections

Over the past two decades, intravitreal injections of anti-vascular endothelial growth factor (anti-VEGF) agents have become increasingly common, particularly in the treatment of neovascular age-related macular degeneration, retinal vein occlusion, and diabetic macular edema. Reported rates of endophthalmitis following intravitreal anti-VEGF injections vary between 0.02% and 0.4% [21,22,23,24,25,26]. Intravitreal corticosteroid injections are performed less frequently but may have a slightly higher rate of endophthalmitis [24]. 

Several factors influence the risks of endophthalmitis following intravitreal injections. Patients taking concurrent systemic immunosuppressive therapy are at increased risk [27]. Additionally, certain injection preparation protocols, such as the use of 2% lidocaine jelly [28], may also increase the risk. The use of sterile pre-loaded compounded formulations from an adequate manufacturing practice pharmacy or manufacturer is associated with a lower infection risk, while physician-initiated self-drawing from medication vials is linked to increased risk [24,29,30]. Systemic preoperative antibiotics do not appear to alter the risk of endophthalmitis following intravitreal injections [31], and various clinical settings, including operating rooms, offices, or hospitals, have generally comparable rates of infection [32]. The use of pre-procedural topical antibiotic prophylaxis is not associated with a reduction in the endophthalmitis rate after injections [22,33,34]. 

## 5. Antimicrobial Resistance Associated with Endophthalmitis

AMR is a global concern that is particularly relevant to the prophylaxis and treatment of endophthalmitis. There is limited evidence regarding ocular microbiome resistance. Understanding ocular microorganism resistance may improve tailored treatments and offer better preoperative, perioperative, and postoperative care decisions, ultimately improving patient outcomes [8].

The most encountered pathogens in ocular infections worldwide include coagulase-negative staphylococci, *Staphylococcus aureus* (*S. aureus*), *Streptococcus pneumoniae* (*S. pneumoniae*), and *Pseudomonas aeruginosa* (*P. aeruginosa*) [35]. The most frequent isolates from acute-onset postoperative endophthalmitis in the US include coagulase-negative staphylococci, *S. aureus,* and streptococci [36]. Streptococcal infections may be particularly severe, due to streptococcal-specific virulence factors including pneumolysin, autolysin, and hyaluronidase, and are often associated with poor visual outcomes, even with prompt and appropriate therapy [37]. 

The eye has potent innate antimicrobial defense mechanisms including mechanical action from repetitive eyelid movements and tear film components including lysozyme, lactoferrin, lipocalin, complement, and secretory immunoglobulin A [38,39]. Penetrating trauma (accidental or surgical) can enable ocular flora to enter and cause infections. Therefore, despite these defenses, ocular surface flora represents a common potential source of the majority of infectious organisms, including *S. aureus*, *Cutibacterium acnes* (formerly *Propionibacterium acnes*), and *Staphylococcus epidermidis* (*S. epidermidis*), among others [2,39,40]. 

Microbes may develop resistance by alteration of the cell wall, modification of surface proteins, direct deactivation of drugs, and acquiring genes and plasmids contributing to resistance [39]. Multi-drug resistant organisms are typically defined as resistant to three or more drugs or three or more categories of antibiotics [40,41,42]. The effects of resistance are potentiated by selective pressures in the community including overuse or misuse of broad-spectrum antibiotics, the use of multiple simultaneous antibiotics or polypharmacy, widespread use for agricultural and veterinary purposes, and improper discarding of medications [6,35,39,42]. In addition, generic antibiotics with suboptimal quality may contribute to AMR [35]. The ocular microbiome has the ability to form biofilms which enhance the ability of pathogens to develop resistance [35]. *P. aeruginosa*, *S. epidermidis*, streptococci, and *Enterobacter* may form biofilms on IOLs, contact lenses, sutures, and various surgical implants, and may lead to a greater number of surgical interventions [39,43,44]. 

The widespread preoperative, intraoperative, and postoperative use of antibiotic therapy in ocular surgeries is a potential source of microbial resistance. Prolonged use of levofloxacin, such as for three weeks or one month after cataract surgery, can lead to levofloxacin-resistant ocular surface flora with a restoration towards sensitive flora after six to nine months after discontinuation. Shorter periods of levofloxacin use, such as one week, can have a faster restoration of susceptibility and lower likelihood of persistently resistant bacteria. The use of some eyedrop preservatives such as benzalkonium chloride (BAC) has been associated with a higher incidence of organisms resistant to methicillin and fluoroquinolones [42]. 

Endophthalmitis is generally treated with empiric broad-spectrum intravitreal antibiotics, which are initiated before culture results can be obtained. Intravitreal agents used in the management of endophthalmitis include glycopeptides such as vancomycin (1.0 mg/0.1 cc), cephalosporins such as ceftazidime (2.25 mg/0.1 cc), or aminoglycosides such as amikacin (0.4 mg/0.1 cc) for suspected bacterial etiologies; and amphotericin-B (0.005 mg/0.1 cc) or voriconazole (0.1 mg/0.2 cc) for suspected fungal etiologies [8]. Forty-eight hours after intravitreal injection, the concentration of vancomycin and ceftazidime in the vitreous are reported to be higher than their minimal inhibitory concentrations (MICs) [45]. Alternatives that have been used for resistant strains include intravitreal clindamycin, linezolid, daptomycin, tigecycline, imipenem, moxifloxacin, ciprofloxacin, and levofloxacin [41].

The use of systemic antibiotics did not provide an added benefit to clinical outcomes in the EVS, although the investigators only studied ceftazidime and amikacin. There are no other published RCTs demonstrating the benefits of systemic antibiotics in the treatment of endophthalmitis. However, several studies have reported on the outcomes of isolated antimicrobials and their use as adjunctive treatment for bacterial endophthalmitis, taking into consideration their penetration of the blood–retinal barrier and the disruption of this layer on patients with endophthalmitis. Some of the most used intravenous antibiotics include meropenem, ceftriaxone, and vancomycin [41]. Of those, enhanced intravitreal therapeutic levels have been reported for meropenem, linezolid, and moxifloxacin. Systemic antibiotics that may achieve effective intravitreal levels include vancomycin, cefazoline, and ceftazidime in aphakic eyes; ceftriaxone after multiple dosing; imipenem and daptomycin in inflamed eyes; and high doses of trimethoprim/sulfamethoxazole [10,41].

AMR in ophthalmology has emerged as a serious public health problem which is difficult to study. The scarcity of available data, primarily due to the low incidence of endophthalmitis, results in a relatively small number of cases for analysis. A clinically useful metric for resistance might be the minimum inhibitory concentration (MIC) of antibiotic that inhibits the growth of 90% of the tested bacterial isolates, or the MIC90, although this metric is not always easy to obtain, and most studies still report traditional resistance rates. Current methods of identifying bacterial susceptibility rely on MICs based on systemic drug use, which may not reflect the higher drug concentrations achieved with intravitreal administration. There is also uncertainty regarding the ocular penetration of antibiotics and their concentrations over time, which may affect clinical effectiveness. In typical clinical ophthalmology practice, infection cultures are seldom acquired, only after initial therapy failure. In such cases, certain organisms may remain untested, potentially biasing reported results towards more severe and resistant infections [10,40]. 

A systematic review of “ocular infections” (sites not always specified) in the US reported high rates of in vitro resistance among *S. aureus* and coagulase-negative staphylococci to fluoroquinolones, macrolides, and methicillin/oxacillin, with high rates of multi-drug resistance [46]. The Ocular Tracking Resistance in US Today (Ocular TRUST) study (2009) reported rates of methicillin resistance in 54% of *S. aureus* isolates and in 62% of coagulase negative staphylococcal isolates (sites not specified) [47]. Another large series of antibiotic testing in the US on cultures obtained from the conjunctiva, cornea, aqueous, and vitreous reported that about one-third of *S. aureus* isolates were resistant to methicillin, one-third were resistant to ciprofloxacin, and almost two-thirds were resistant to azithromycin [48]. Metallo β-lactamase—which inhibits the action of β-lactam antibiotics—may be produced by gram-negative bacteria isolated from ocular infections, especially in *P. aeruginosa* showing resistance to cephalosporins and meropenem [49]. 

Since 2009, the Antibiotic Resistance Monitoring in Ocular Microorganisms (ARMOR) study has tracked the in vitro resistance of ophthalmic microorganisms. Recent findings from the study, with a subset of isolates from the aqueous and vitreous in endophthalmitis cases, reported concerning resistance trends. Coagulase-negative staphylococci and *S. aureus* displayed high resistance rates, with over 45% resistant to methicillin, over 57% resistant to azithromycin, and over 44% resistant to ciprofloxacin. Notably, more than 70% of staphylococci isolates exhibited multi-drug resistance. Additionally, *S. pneumoniae* showed resistance rates of over 38% for azithromycin and penicillin, and *P. aeruginosa* demonstrated 100% resistance to polymyxin B [50,51,52]. However, gram-positive organisms maintained 100% susceptibility to vancomycin in both the ARMOR and EVS reports [53,54,55].

Among gram-negative bacteria, approximately 90% showed sensitivity to both amikacin and ceftazidime. Nevertheless, certain gram-negative pathogens such as *P. aeruginosa* have developed resistance to broad-spectrum antibiotics, including fluoroquinolones, amikacin, and ceftazidime [8,56]. Notably, a recent multistate outbreak associated with drug-resistant *Pseudomonas aeruginosa* linked to artificial tears eye drops resulted in severe cases of keratitis, panophthalmitis, vision loss, and even enucleation and death [57,58,59,60]. Globally, reports have indicated a rising trend in resistance to fluoroquinolones and ceftazidime [61,62,63]. There were also differences in the minimum inhibitory concentrations (MIC90s) among fluoroquinolones, with consistently lower levels found with newer-generation fluoroquinolones, such as besifloxacin [48]. 

These are FDA-approved for serious infections with multidrug-resistant gram-negative bacteria, especially those caused by Enterobacteriaceae, *Pseudomonas aeruginosa* and *Acinetobacter baumannii*. Polymyxins are often the only effective antibiotic agent against multidrug-resistant organisms, particularly carbapenem-resistant Enterobacteriaceae.

Pediatric data have indicated antibiotic resistance in infections secondary to staphylococci or pneumococcal organisms, and similar multidrug resistance among staphylococci [64]. Local and regional US studies have also showed similar resistance trends, with a peak methicillin-resistant *S. aureus* (MRSA) prevalence prior to 2015, with a slight decreasing trend afterwards [48]. In the context of endophthalmitis, various studies have reported that patients with gram-positive samples including *Enterococci* were strongly susceptible to vancomycin, while gram-negative isolates showed full to intermediate susceptibility to ceftazidime, highlighting intravitreal use of these broad-spectrum medications as a first-line treatment [65,66,67]. Some reports have shown concerning cases with vancomycin resistance [9,68,69,70]. Among patients with gram-positive cultures resistant to vancomycin, linezolid or daptomycin have been used as alternative treatments [71,72]. Among those with gram-negative cultures displaying resistance to ceftazidime, certain individuals exhibit sensitivity to ciprofloxacin, moxifloxacin, and imipenem [56,73]. 

When reviewing drug resistance rates in ophthalmic diseases, it is important to consider that very high concentrations of antibiotics may be delivered to infected tissues, such as topical antibiotics for ocular surface infections or intravitreal antibiotics for endophthalmitis. Many bacteria with reported in vitro resistance may respond well to clinical therapy. Therefore, certain patients infected with “resistant” organisms may experience a favorable clinical response, which should serve as the primary factor guiding decision-making in their management [41,74,75]. Similarly, an absence of improvement within the initial 48–72 h should raise suspicion about the presence of a drug-resistant organism, even in the absence of laboratory sensitivity results [41].

## 6. Current Prophylactic Measures and Management Approaches

Patients with endophthalmitis may have very unfavorable clinical outcomes in spite of prompt diagnosis and proper therapy. This highlights the importance of prophylaxis. Endophthalmitis cannot be “prevented” but its incidence can be reduced using various techniques. Prophylaxis includes more than the use of antibiotics; it encompasses preoperative patient evaluation, the surgical “prep”, antisepsis, and meticulous surgical techniques in addition to the judicious administration of antimicrobial agents. Important steps include antisepsis with topical povidone-iodine, hand hygiene, sustaining a sterile procedure field, and a highly selective protocol for the use of pre- and postoperative prophylactic antibiotics [75].

The goal of preoperative antisepsis is to minimize the pathogen load in the conjunctiva and eyelids, since ocular flora is the most common source of infection [76]. The use of preoperative povidone-iodine antisepsis is well established [77,78]. Povidone-iodine has broad-spectrum antimicrobial activity against bacteria, fungi, protozoa, and viruses and has limited reported adverse effects. It is inexpensive and has a rapid onset of action, with effectiveness starting as soon as one minute upon skin contact. It also decreases bacterial growth from the conjunctiva without inducing antibiotic resistance, achieving a significant reduction in culture positivity rates [78,79,80]. The recommended povidone-iodine concentration varies. Numerous studies have supported the use of 5% povidone-iodine on the ocular surface [81,82]. The European Society of Cataract and Refractive Surgery (ESCRS) recommends 5–10% povidone-iodine prior to cataract surgery, with an alternate of 0.05% aqueous chlorhexidine in case of iodine allergy or hyperthyroidism [81,82]. The American Academy of Ophthalmology (AAO) also recommends 5% povidone-iodine but does not recommend chlorhexidine due to reports of corneal surface toxicity causing irreversible keratitis [83,84]. 

In a RCT comparing povidone-iodine 5%, polyhexanide biguanide (PHMB) 0.02%, and chlorhexidine 0.02% in cataract surgery, there were no significant differences between the three agents in reducing the numbers of colony-forming units [85]. Another RCT reported no additional efficacy with adding topical 0.5% moxifloxacin to 5% povidone-iodine [86].

The prophylactic use of antibiotics perioperatively to reduce endophthalmitis remains controversial and uncertain and varies geographically around the world. The AAO and the ESCRS have both reported that the use of preoperative topical antibiotics is unnecessary and not cost-effective [87]. Postoperatively, there were no differences in prophylaxis efficacy among various classes of topical antibiotics, including gatifloxacin, ofloxacin, and polymyxin/trimethoprim. However, topical postoperative aminoglycosides were reported to be ineffective [88]. There has been a trend of decreasing utilization of postoperative topical antibiotics [89,90,91]. Techniques such as intraoperative injections offer the benefit of assured delivery of drugs and avoidance of patient-related complications. Patient-dependent topical administration of eye drops carries the risk of poor patient compliance, microtrauma to eye, contamination of the bottle, and insufficient or prolonged administration of drops, which carry a risk of AMR [89].

There is an increasing use of routine prophylactic intracameral antibiotics around the world [92]. The ESCRS published a RCT of over 16,000 patients, randomized to receive intracameral cefuroxime 1 mg/0.1 mL with or without topical levofloxacin, and reported that intracameral cefuroxime was associated with an approximate five-fold reduction in the rate of endophthalmitis after cataract surgery [93]. Another RCT studied intracameral 0.5% moxifloxacin and reported that intracameral moxifloxacin was associated with a reduced rate of endophthalmitis [94]. In addition, many retrospective series and prospective studies have reported benefits with intracameral antibiotic prophylaxis in cataract surgery [88,95,96,97,98,99,100,101,102,103,104]. Further, several meta-analyses have also supported the use of intracameral antibiotics, highlighting a greater effectiveness of this route as a prophylactic measure and a lack of additional benefits from topical antibiotic administration [100,105,106]. Although intraoperative complications such as posterior capsular rupture and vitreous loss are associated with a more than three-fold increase in endophthalmitis, intracameral antibiotics have demonstrated marked efficacy as prophylaxis in these complicated cases [107,108,109]. 

There are several concerns regarding these studies. Cefuroxime is a second-generation cephalosporin mainly active against gram-positive organisms but does not provide coverage against gram-negative bacteria and is ineffective against MRSA and enterococci. Additionally, the effectiveness of intracameral cefuroxime is time-dependent with concentrations above the MIC only for about 5 h, which suggests a short-term benefit [110]. Vancomycin is very effective against gram-positive organisms, including MRSA, but less effective against common gram-negative organisms. Intracameral vancomycin remains above the MIC for about 32 h after administration [111]. Moxifloxacin is a fourth-generation fluoroquinolone that has a broader spectrum against both gram-positive and gram-negative isolates and is able to achieve a greater concentration than the MIC for a longer time than cefuroxime. The effectiveness of this medication is dose-dependent, and there have been reports of bacterial resistance to moxifloxacin being overcome by administering higher, yet safely tolerable, doses [20,112,113]. 

Although intracameral antibiotics have the benefit of greater intraocular concentrations, their use is not innocuous [114]. Preservative-free intracameral moxifloxacin is associated with decreased corneal cell density and increased apoptotic markers of the cornea as well as two case reports of uveitis [100,108,115,116,117,118,119]. Intracameral cefuroxime also seems to have a favorable safety profile, but overdoses due to compounding mistakes have been associated with uveitis, macular and corneal edema, and toxic anterior segment syndrome, and patients with penicillin allergies may have an anaphylactic reaction [100,118,119,120]. Vancomycin use has been associated with ischemic retinal vasculitis and hemorrhagic occlusive retinal vasculitis (HORV) [121,122,123].

In response to a 2021 survey from the American Society of Cataract and Refractive Surgery (ASCRS), about 66% of respondents reported the prophylactic use of intracameral antibiotics, representing an increasing trend from previous reports published in 2014 (50%) and 2007 (30%). Moxifloxacin was the most commonly used, as reported by 83% of the respondents [92]. Of those not using intracameral antibiotics, a majority responded that they would use an affordable and approved product if one were available in the US [92]. Currently, there is a packaged formulation of cefuroxime approved in Europe (Aprokam^®^, Laboratories Théa, Clermont-Ferrand, France). In the US, there is no approved medication and no universally accepted dose of “off-label” medication, although 500 μg of the preservative-free formulation of moxifloxacin (Vigamox^®^, Alcon, Fort Worth, TX, USA) is widely used [124,125]. 

An approved, affordable product would increase utilization in the US, as has occurred in India with intracameral moxifloxacin [126]. Another proposed technique is to use an IOL loaded with antibiotics (moxifloxacin, gatifloxacin) and anti-inflammatories (ketorolac). The latter would allow a sustained and extended drug release with the challenge of avoiding a negative effect on the lens’ physical properties or on the surrounding ocular tissues [127,128,129,130,131,132]. 

Most patients only undergo cataract surgery once per eye per lifetime, so the cumulative risk of endophthalmitis after cataract surgery is limited. However, intravitreal injections are generally performed repeatedly over a period potentially lasting many years. This cumulative risk may be mitigated by using newer intravitreal medications with longer durations, thus requiring fewer injections. These include newer anti-VEGF agents faricimab (Vabysmo^®^, Genentech, South San Francisco, CA, USA) and high-dose aflibercept (Eylea HD^®^, Regeneron, Tarrytown, NY, USA); the dexamethasone delivery system (Ozurdex^®^, Allergan, Inc., Irvine, CA, USA); and the fluocinolone acetonide intravitreal insert (Iluvien^®^, Alimera Sciences, Alpharetta, GA, USA) [133].

## 7. Stewardship in Ophthalmology

Antibiotic stewardship initiatives promote a collaborative, multidisciplinary approach aimed at fostering the judicious use of antimicrobial agents. Key strategies to reduce antimicrobial resistance are described in this section and summarized in Table 1. The primary goal is to ensure that antibiotics are prescribed correctly, including selecting the appropriate drug and determining the correct dosage, route, and treatment duration in relation to local resistance patterns. Such programs have been reported to mitigate the emergence of resistant organisms and reduce healthcare costs without compromising clinical outcomes [134,135,136,137]. Stewardship programs depend on awareness, research, policies, and targeted (rather than widespread and broad-spectrum) antibiotic use [35]. 

The US CDC has outlined the Core Elements of Outpatient Antibiotic Stewardship to provide a framework for antibiotic stewardship for healthcare departments and outpatient clinicians that routinely provide antibiotic treatment. The key elements emphasize leadership commitment, accountability, stewardship expertise, action for policy and change, tracking, reporting, and education in acute care and long-term care settings. The CDC actively tracks data on outpatient antibiotic prescriptions from various sources to gain insights into prescribing patterns, identify areas requiring interventions, and gauge improvement. The most recent report indicates that, as of 2022, 97% of hospitals have successfully implemented all Core Elements [138]. Stewardship programs have led to a decrease in infections caused by drug-resistant pathogens, along with 18% fewer deaths from antibiotic resistance overall [1].

Specific stewardship recommendations stipulate that the concentration of antibiotics must be greater than or equal to the MIC and preferably the minimum bactericidal concentration at the infection site, as using lower concentrations than the MIC90 may select for resistant organisms. Other recommendations also emphasize the use of appropriate dosages and durations of therapy and the limiting of the use of multiple agents [35]. Empiric broad-spectrum treatment is useful in many situations (such as in the initial management of most patients with endophthalmitis), but, ideally, the antibiotic regimen will be de-escalated and tailored as diagnostic culture results become available. In empiric treatment, the option of combination therapy can be considered to improve therapeutic efficacy or expand the range of targeted pathogens [35,139]. 

Antibiotic stewardship programs have been successfully implemented in various healthcare settings, but they have not been widely adopted within ophthalmology. There are no specific guidelines for antimicrobial prescription in ophthalmology which result in widely used treatments for minor and self-limiting conditions as well as pre- and postsurgical prophylaxis [136]. The principles of stewardship suggest that, ideally, antimicrobials should be used only when medically needed to treat established infections and not for prophylaxis. However, the specific agents, doses, durations, and clinical indications are generally undetermined in eye conditions [136]. 

The use of routine prophylactic intracameral antibiotics contradicts the principles of antibiotic stewardship [41]. It has been recommended not to prescribe fluoroquinolones as monotherapy since this is associated with increased risks of selecting for resistant organisms and encouraging MRSA colonization [140,141]. As a potential mitigating factor, the high concentrations of medications instilled via intracameral administration usually exceed the MIC, which reduces the risk of drug resistance [141]. The use of intracameral vancomycin defies multiple CDC recommendations to reserve the use of vancomycin for life- or organ-threatening infections, rather than for prophylaxis [142,143]. Vancomycin remains the most important agent against gram-positive organisms in patients with established endophthalmitis, so the use of vancomycin for prophylaxis undermines its effectiveness by promoting resistant organisms [122,123]. 

The ideal broad-spectrum antibiotic is yet to be established. AMR is an increasing worldwide clinical challenge. One possible strategy involves the use of known antimicrobial agents that develop resistance less frequently due to the specificity of their mechanism of action. One example is polymyxins, which have demonstrated efficacy against multi-drug resistant gram-negative organisms. However, the safety and efficacy of intravitreal polymyxins remain unestablished at this time [53,54,55]. 

When evaluating any intracameral antibiotic, one should consider a number-needed-to-treat analysis, which represents the average number of patients who must be exposed to the antibiotic in order to “prevent” one case of endophthalmitis. Antisepsis is clearly more cost-effective than antibiotic treatment [41]. 

Future advancements in the field may explore the utilization of bacteriophages and antimicrobial peptides to combat bacterial ocular infections, the integration of hydrogels in contact lenses for biofilm prevention, the development of liposomal-lactoferrin-based eye drops to diminish pathogen presence, and the implementation of gene-targeting strategies to address antimicrobial resistance [144,145,146,147,148,149]. There is also ongoing research exploring the potential of stem cells as adjuncts to antimicrobial treatments to aid in the repair of damaged ocular tissues and combating septic infections [150].

Additionally, efforts are being made to investigate plant-based methods and natural products as potential solutions to antimicrobial resistance. It has been proposed that naturally occurring compounds (including plant metabolites), rather than fully synthetic molecules (such as sulfonamides, fluoroquinolones, and oxazolidinones) would be better suited to overcome AMR [151,152]. However, none of these strategies are in current widespread clinical use.

Guidelines for the judicious use of antibiotics include monitoring and providing specific feedback regarding prescribing patterns specific to different ocular infections [35,41,136]. Large scale surveillance for ocular infections is important. Active surveillance programs in the United States gather country and world-wide data to track drug resistance in ophthalmology through the Ocular Tracking Resistance in the U.S. Today (TRUST) and ARMOR programs [40,141]. Promoting such programs may aid studying the local patterns of AMR and in allowing effective implementation of antibiotic stewardship programs both nationally and globally [35,153].

## 8. Limitations 

The literature search and subsequent review were limited to English-language publications. While this was undertaken to uphold the accuracy of information within this review, it may have inadvertently omitted valuable insights and nuances regarding antimicrobial resistance strategies documented in non-English-language countries. Furthermore, as this review adopted a standard literature review approach rather than a systematic methodology, it was constrained by the absence of rigorous systematic methods and statistical analyses, potentially introducing bias and limiting the reliability of the conclusions by not providing a quantitative summary of the evidence.

## 9. Conclusions

The rise of AMR in endophthalmitis is a global concern, necessitating a comprehensive approach. Antibiotic stewardship programs are more robust in other medical specialties than in ophthalmology. Developing a better understanding of the ocular microbiome, ophthalmic infections, and defining region-specific bacterial susceptibility and resistance trends, is important for tailoring treatments and improving patient outcomes. Reporting on current prescription trends, emergence of multi-drug resistant organisms, and promoting antimicrobial stewardship programs is key to addressing AMR in ophthalmology effectively. 

## Figures and Tables

**Table 1:** KEY STRATEGIES TO REDUCE ANTIMICROBIAL RESISTANCE.

***CLINICAL STRATEGIES***
Strict adherence to sterile surgical protocols.
Use of povidone-iodine as an antiseptic.
Minimizing polypharmacy when feasible.
Obtaining early culture samples in cases of clinically suspected infection.
Tailoring antibiotic therapy based on culture results and de-escalation of antibiotic regimen.
Avoiding long-term use of antimicrobials.
Reducing the prophylactic use of antimicrobials in uncomplicated procedures.
Developing specific guidelines for antimicrobial use in ophthalmic conditions to promote evidence-based prescribing practices.
***PUBLIC HEALTH STRATEGIES***
Identifying region-specific bacterial susceptibility and local antimicrobial resistance patterns for ocular infections.
Analyzing current prescription trends (from eye-care providers, primary physicians, and pharmacies) to identify areas for intervention.
Investing in research for alternative non-antibiotics antimicrobial strategies (bacteriophages, antimicrobial peptides, gene-targeting strategies).
Establishing antimicrobial stewardship programs.
Educating patients about the importance of completing prescribed antimicrobial courses, avoiding self-medication, and adhering to hygiene practices to help in preventing the spread of resistant strains.
Encouraging global collaboration to implement effective antibiotic stewardship programs and combating antimicrobial resistance worldwide.

## Data Availability

Not applicable.
